# The Dark Side of Precision: Pin-Related Complications in Computer-Navigated and Robotic-Assisted Knee Arthroplasty

**DOI:** 10.3390/jcm15103793

**Published:** 2026-05-14

**Authors:** Gabriele Di Carlo, Biagio Zampogna, Natale Criseo, Domenico Aragona, Oriana Pugliesi, Salvatore Calaciura, Domenico Fenga, Ilaria Sanzarello, Danilo Leonetti

**Affiliations:** 1 BIOMORF Department of Biomedical, Dental, Morphological and Functional Images, University of Messina, Orthopaedic and Trauma Surgery, A.O.U. Policlinico “G.Martino”, Via Consolare Valeria 1, 98124 Messina, Italy; gabriele.dicarlo@polime.it (G.D.C.); biagio.zampogna@unime.it (B.Z.); natale.criseo@polime.it (N.C.); domenico.aragona97@gmail.com (D.A.); orianapug@hotmail.it (O.P.); dott.calaciurasalvatore@gmail.com (S.C.); domenico.fenga@polime.it (D.F.); ilaria.sanzarello@unime.it (I.S.); 2 Research Unit of Orthopaedic and Trauma Surgery, Fondazione Policlinico Universitario Campus Bio-Medico, 00128 Rome, Italy; 3 Fondazione Policlinico Universitario Campus Bio-Medico, Via Alvaro del Portillo 200, 00128 Rome, Italy

**Keywords:** pin, complication, knee arthroplasty, computer navigation, robotic

## Abstract

**Background:** With the rising volume of knee arthroplasty and increasing adoption of robotic- and computer-assisted systems, the routine use of tracker pins has introduced procedure-specific risks. This systematic review aimed to characterize the types and incidence of pin-site complications associated with robotic-assisted and computer-navigated primary knee arthroplasty and to describe the timing, management strategies, and reported outcomes. **Methods**: A PRISMA-guided search of PubMed/MEDLINE was performed using terms related to pin-related complications, robotic assistance, computer navigation, total and unicompartmental knee arthroplasty procedures. Clinical studies (RCTs, cohorts, case series, and case reports) that explicitly documented pin-related complications in robotic- or computer-assisted knee arthroplasty in English were included. Two independent reviewers performed study selection and data extraction; the methodological quality of non-randomized studies was assessed with the MINORS instrument. Extracted variables encompassed study design, patient demographics, pin characteristics, type and timing of complications, treatments, and outcomes. Descriptive statistics and means were used where appropriate. **Results**: From 1231 initial records, 28 studies met the inclusion criteria, comprising 15,004 cases in cohort/series analyses. The aggregate pin-related complication incidence in non-case-report series was 0.95% (142 events). Of these, 13.4% were intraoperative and 86.6% postoperative. The most common postoperative events were pin-site wound issues and infections (each ≈35.7% of complications); pin-site fractures accounted for 0.16% in cohort/series data. Case reports (n = 17 patients) showed fractures chiefly at femoral pin sites, arising on average 8.5 weeks postoperatively; management ranged from protected weight-bearing to intramedullary nailing or ORIF. Potential risk factors suggested in the literature include higher BMI, bicortical or transcortical fixation, metaphyseal pin placement, and larger pin diameter, but findings were inconsistent. **Conclusions**: Pin-related complications after robotic- and computer-assisted knee arthroplasty are uncommon but clinically significant (≈0.95%). There is insufficient evidence to define optimal pin-placement strategies or fixation configurations. Surgeons should include pin-related risks in informed consent discussions. Further prospective research is required to identify patient- and technique-specific risk factors and to establish evidence-based pin-placement guidelines.

## 1. Introduction

By 2050, the annual number of total knee arthroplasty (TKA) procedures performed in the United States is projected to reach approximately 1.5 million, representing an increase of about 143% compared with 2012 [1]. Similarly, increasing trends have been reported for unicompartmental knee arthroplasty [2,3].

In parallel, the use of robotic-assisted and computer-assisted techniques in knee arthroplasty is steadily increasing and is expected to continue to expand over the coming years.

The utilization of robotic arm-assisted technology in total knee arthroplasty (TKA) has increased dramatically in recent years, rising from less than 0.1% of procedures in 2008 to 4.3% in 2018. Should this trajectory persist, projections suggest that by 2030, approximately half of all TKAs performed in the United States could be carried out with robotic assistance [4,5].

Robotic-assisted and computer-assisted knee arthroplasty have been introduced with the aim of improving the precision and reproducibility of bone resections and component positioning. Multiple studies have demonstrated that these technologies can reduce the proportion of radiographic outliers in coronal and sagittal alignment, improve restoration of the mechanical or kinematic axis, and allow for more consistent soft-tissue balancing compared with conventional techniques [6,7,8,9].

Systematic reviews and meta-analyses suggest that such improvements in accuracy may be associated with reduced early postoperative pain, faster early functional recovery, and, in some cohorts, modest gains in patient-reported outcome measures, although the magnitude and durability of these clinical benefits remain debated [5,10,11,12].

However, the introduction of robotic and navigation systems also adds layers of complexity and creates specific risks that are not present, or are far less common, in conventional arthroplasty [10,13].

These include issues related to workflow, learning curve, increased operative time, capital and maintenance costs, and—crucially for the present review—device- and technique-specific complications.

Among these, pin-related complications occupy a central role. Most optical, robotic, and navigation platforms require temporary percutaneous or intra-incisional insertion of tracking pins into the femur and tibia to secure reference arrays or sensors. While pin placement is usually straightforward, it introduces stress risers and additional skin and soft-tissue breaches and may therefore act as a source of adverse events [14,15,16].

Pin-related complications described in the literature include superficial and deep pin-site infection, delayed wound healing, persistent pain, neurovascular injury and intraoperative or postoperative fractures through the pin tracts [14,15,16]. Recent systematic reviews on computer-navigated and robotic knee arthroplasty have reported overall pin-site complication rates typically between 0.3% and 1.9%, with superficial infection and drainage being the most frequent events [14,16]. Nonetheless, periprosthetic fractures through tracking pin sites, although rare—with incidences in larger series on the order of 0.16–0.3%—represent serious complications that can necessitate restricted weight bearing, osteosynthesis, or even revision arthroplasty, and can significantly compromise functional outcomes [15,16,17].

Given the projected increase in the number of robotic-assisted and computer-assisted knee arthroplasty procedures, we sought to conduct a systematic review of the literature with the following objectives: (1) determine the type and incidence of pin-related complications associated with robotic-assisted and computer-navigated primary knee arthroplasty; (2) describe the timing of complications reported, treatment strategies adopted and reported outcomes.

## 2. Materials and Methods

The Preferred Reporting Items for Systematic Reviews and Meta-Analyses (PRISMA) guidelines were followed, and a flowchart was created to summarize the inclusion process of the analyzed works. A detailed protocol for this systematic review was not registered in any public registry. The PRISMA checklist has been duly completed (Supplementary Material).

The medical PubMed/MEDLINE databases were analyzed on 10 November 2025, searching for relevant publications on pin site-related complications. The databases were filtered for studies published between January 1995 and November 2025, in English. The keywords used for the search were “pin related complications”, “robotic assisted”, “computer navigated”, “total knee arthroplasty”, “total knee replacement”, “unicompartmental knee replacement”, “unicompartmental knee arthroplasty”. These terms were combined into the following search string: (((((((((pin site fracture) AND (pin site infection)) AND (pin site complication)) AND (robotic total knee arthroplasty)) OR (robotic total knee replacement)) OR (robotic UKA)) OR (Robotic UNI)) OR (robotic unicompartmental knee)) NOT (total hip arthroplasty)) NOT (outcome).

To maximize the capture of relevant evidence, we screened the reference lists of included studies and relevant reviews.

Studies were deemed eligible for inclusion if they satisfied all of the following criteria: (1) clinical design (randomized controlled trial, cohort study, case series, or case report) evaluating the use of computer-assisted navigation (CAN) or robotic assistance (RA) in total or partial knee arthroplasty; (2) explicit documentation of pin-related complications associated with CAN or RA techniques; and (3) publication in English.

The following were excluded: biomechanical and cadaveric studies, expert opinions, commentary letters, review articles, meta-analyses, and textbook chapters. Studies that reported no pin-related complications or that only speculated on potential pin-related events—without clinical, radiographic, or other diagnostic confirmation—were excluded. Furthermore, studies published in a language other than English were excluded.

The titles and abstracts of the identified papers were analyzed and screened independently by two authors. The articles judged to be of interest were then selected for full-text analysis according to our inclusion and exclusion criteria. Any disagreement between reviewers during each step of the review process was resolved by discussion between the two reviewers. If a consensus could not be reached, final inclusion was decided by a senior reviewer (D.L.).

The level of evidence and methodological quality of each included study were independently appraised by two reviewers using the Methodological Index for Non-Randomized Studies (MINORS) and the RoB 2.0 tool for randomized studies [18,19]. The MINORS comprises 12 items, of which the first eight are specifically designed to assess the quality of non-randomized research. These core items address whether the study objective is clearly defined, whether patients are enrolled consecutively, whether data collection is prospective, and whether the primary endpoint is both appropriate to the stated aim and assessed in an unbiased manner. In addition, MINORS evaluates whether the duration and reporting of follow-up are adequate for the study question, with an acceptable loss to follow-up defined as less than 5% of the initial cohort, and whether a priori sample size calculation is explicitly described in the methods.

Each item is scored on a three-point scale: 0 if not reported, 1 if reported but inadequate, and 2 if reported and adequate. For non-comparative studies, the maximum attainable (optimal) MINORS score is 16.

After final exclusions, standardized data extraction was performed independently by two reviewers.

Relevant data were extracted into a database created in Microsoft Excel for Mac (Microsoft Corporation, Redmond, WA, USA).

For each included study, the following variables were collected, if available: study design, study period, institution, patient demographics (age, sex, body mass index [BMI], comorbidities, and length of follow-up), CAN/RA brand and model, pin trajectory, cortical penetration, pin location, pin diameter, number of pins, pin-related complications, incidence of pin-related complications, timing of complications (intraoperative vs. postoperative), time to postoperative complications, anatomical location of complications (femur vs. tibia; diaphysis vs. metaphysis), treatments administered and clinical outcomes.

Descriptive statistical analyses were performed for each included study. For continuous variables, weighted means and standard deviations were calculated based on the number of subjects in each individual study.

## 3. Results

The initial search yielded 1231 articles for review. After screening for duplicate citations, 1181 articles remained. After screening for appropriateness based on title and abstract, 1137 studies were excluded.

**Figure 1:**
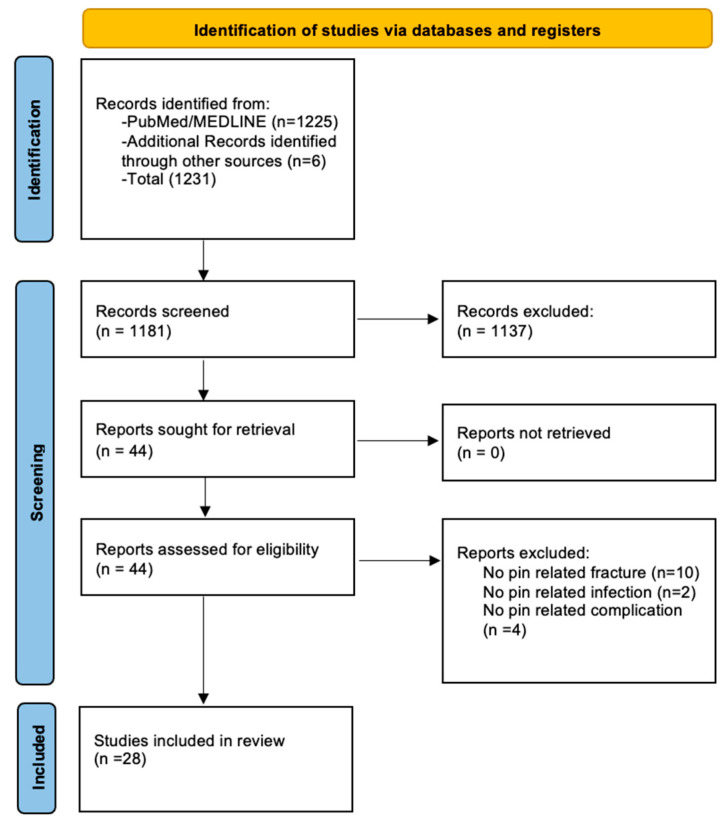
PRISMA flowchart.

The remaining 44 articles were examined in full to assess their compliance with the predefined inclusion criteria. Following this full-text assessment, 28 studies were deemed eligible for inclusion (Figure 1). Table 1 summarizes the characteristics of the included studies.

Of the included studies, one was a randomized controlled trial (Level II Evidence) [19], 6 were retrospective cohort studies (Level III Evidence) [20,21,24,26,28,31], 1 was a prospective cohort study (Level IV Evidence) [22], 1 was a prospective case series (Level IV Evidence) [32], 6 were retrospective case series (Level IV Evidence) [14,23,25,27,29,30] and 13 were case reports (Level V Evidence) [17,33,34,35,36,37,38,39,40,41,42,43,44].

Manuscripts were published between 2006 and 2025.

According to the selected score system, the mean MINORS score was 13.7 for the included non-randomized studies (12 of 16 for non-comparative studies and 15.5 of 24 for comparative studies).

The randomized trial by Todesca et al. [19] was appraised using the RoB 2.0 [45] tool and received an overall risk-of-bias judgment of “Low”.

### 3.1. Case Reports

Demographic and surgical details of the case reports included in this study are illustrated in Table 2.

The cohort consisted of a total of 17 patients from 13 studies included in the database, of whom 15 were female (88.2%) and 2 male (11.2%), with a mean age of 66.5 years (range 46–77 years).

BMI was reported for 9 patients [17,33,34,38,39,40,42], with a mean value of 33.1 kg/m^2^ (range 20.8–42 kg/m^2^).

The mean postoperative follow-up, expressed in months, was 9.95 months (range 1.5–36 months), calculated on 10 out of 17 patients.

Among the 17 patients, 5 were treated with RA techniques [33,39,41,42] and 12 with CAN techniques [17,34,35,36,37,38,40,43,44].

Four studies [34,38,41,43] reported associated comorbidities, including osteoporosis treated with Denosumab associated with multiple fragility fractures [41], arterial hypertension (2 cases) [38,43], irritable bowel syndrome [34], hyperlipidemia [38], chronic kidney disease [38], and obesity.

Only one case report [35] mentioned surgeon experience, described as “senior surgeons”.

Table 3 lists complications related to pin use, their management, and the corresponding outcomes.

The most frequent complication was fracture related to pin use, observed in 14 out of 17 patients (82.4%), including 2 stress fractures [40,44]. All fractures (100%) occurred postoperatively, with a mean time to onset of 8.5 weeks (range 4–12 weeks).

Four fractures were minimally displaced or nondisplaced stress fractures (28.57%), while the remaining 10 were displaced oblique or transverse fractures (71.43%).

Twelve fractures occurred at the femoral pin insertion site (85.7%), whereas the remaining two occurred at the tibial pin insertion site (14.28%).

Eight fractures (57.14%) were treated with antegrade or retrograde intramedullary nailing, 2 (14.28%) were treated conservatively by avoiding weight-bearing on the affected limb (stress fractures), 3 (21.42%) were treated with open reduction and plate fixation, and 1 (7.14%) was managed with combined intramedullary nailing and plate fixation.

Of the 14 fractures, 8 (57.14%) were associated with bicortical pin fixation, 1 (7.14%) with unicortical fixation, 2 (14.28%) with erroneously performed transcortical fixation instead of bicortical fixation, while in the remaining 3 cases the fixation method was not specified.

The remaining 3 complications (17.65%) included myositis ossificans [34], occurring at the femoral pin site 2 weeks postoperatively and treated with open mini-arthrotomy excision; Staphylococcus aureus osteomyelitis [36], occurring 2 weeks and 5 days after surgery at the tibial pin site and initially treated with antibiotics, followed by recurrence at 6 months requiring surgical opening, washout and debridement, and long-term antibiotic therapy; and a hematoma [37] detected 3 days after surgery but resulting from intraoperative bleeding within the femoral canal not immediately recognized and originating from the femoral pin site, treated on postoperative day 10 with hematoma evacuation and placement of two unicortical screws to tamponade bleeding. Residual stiffness was subsequently treated with manipulation under anesthesia. All these other complications had been resolved at final follow-up without further sequelae.

### 3.2. Randomized Trial, Case Series and Cohort Studies

Fourteen of the fifteen included studies (93.3%) were single center. In ten studies, the procedures were performed by experienced surgeons; in one study by a surgeon performing at least 20 RATKA procedures per year [26]; in one study by a group of surgeons with heterogeneous levels of experience [23]; and in one study by a surgeon without prior robotic surgery experience [32]. In two studies, the surgeons’ experience level was not reported [21,30].

A total of 15,004 cases (including TKA, UKA, and PFA) were analyzed; the mean age was 66.8 years, and 61.03% were female. The mean BMI in studies reporting this value was 30.1 kg/m^2^. Specific comorbidities of patients who experienced pin-related complications were not reported. Mean follow-up was 15.7 months (range 2–77 months).

Among a total of 15,004 included cases, 142 pin-related complications, occurring in 142 patients, were recorded (0.95%); of these, 19 (13.38%) were intraoperative and the remaining 123 (86.62%) were postoperative.

The most frequent intraoperative pin-related complication was pin breakage (7 of 19; 36.84%) and pin-site fracture (7 of 19; 36.84%). Five cases of pin dislodgement were recorded (26.32%). Thirteen of the 19 intraoperative complications (68.42%) involved the tibia, one (5.26%) involved the femur, and five (26.32%) did not have the site specified.

In two of the seven cases of pin breakage, the robotic procedure was continued; management was not reported for the remaining five cases. In all five cases of pin dislodgement, the robotic procedure was aborted. In cases of intraoperative fracture, postoperative management was not altered.

Among postoperative complications (n = 123), the most frequent events were 44 pin-site wound complications (35.77% total; 41 tibial 93.2%, 3 femoral 6.8%); 44 pin-site infections (35.77% total; 40 tibial 90.9%; 4 unspecified, 9.1%); 17 pin-site fractures (13.82% total; 14 femoral, 77.8%; 4 tibial, 22.2%). Of the 17 fractures, 12 (70.59%) occurred with bicortical pin fixation (of which 5 cases had transcortical pin placement), 2 (11.7%) with monocortical fixation, and in 3 cases (16.65%) the pin fixation method was not described. Other complications described included 4 cases of delayed wound healing (3.25% total; 2 tibial, 50%; 1 femoral, 25%; 1 unspecified, 25%); 5 cases of tibial pin-site erythema or drainage (4.07%); 2 cases of heterotopic ossification (1.63%, site not specified); 1 case of tibial osteomyelitis (0.81%); 1 tibial suture-site abscess (0.81%); 4 cases of pin-site irritation (3.25%, site not specified); and 1 pseudoaneurysm of the anterior tibial artery (0.81%).

Patients’ demographic and surgical details are summarized in Table 4. Pin-related complication treatments and outcomes where reported are summarized in Table 5.

## 4. Discussion

Parallel to the increase in knee arthroplasty procedures, there has been a rise in the use of robot- and computer-assisted prosthetic systems [1,46]. These technologies offer recognized advantages in the precision of preoperative planning, execution of bone cuts, and implant alignment, which appear to translate into improved outcomes, shorter hospital stays, and reduced analgesic consumption [1,47,48,49,50]. However, the use of such systems requires the placement of trans-osseous pins, which may be associated with specific risks inherent to these procedures. The aim of this comprehensive review is to present the potential complications related to pin placement, their management, and, where reported, their impact on clinical outcomes.

From the analysis of the 15 non-case-report studies included out of 28 studies, the rate of pin-related complications was 0.95%, lower than the 1.4% reported by Thomas et al. [15]. Of these complications, 13.38% occurred intraoperatively and 86.62% occurred postoperatively.

The most common intraoperative complication was pin breakage (7 cases; 36.8% of intraoperative events). In two of these cases the robotic procedure was continued; in the remaining five cases no management details were reported. Regarding pin-site fracture (7 cases; 36.8%), no modification of the normal postoperative course was reported. In the five cases of pin dislodgement (26.3%) the robotic procedure was interrupted.

Evaluating postoperative pin-site complications, the most frequent were generically reported as “pin-site wound complications” and “pin-site infections,” each representing 35.7% of complications. Additionally, 17 pin-related fractures were observed (13.82%).

Among soft-tissue pin-related complications (incidence 0.59%), over 90% on average were recorded at the tibial site.

No patient-specific risk factors were identified; however, Khakha et al. [27] hypothesized that the increased tibial risk is related to the superficial anatomical position of the tibia and tenuous soft tissue coverage. In any case, these superficial complications do not appear to be associated with an increased risk of deep infection.

It is noteworthy that among eight studies reporting superficial wound and infectious complications related to pin placement, femoral pins were placed through the main incision in 50% of cases; one study evaluated two pin-placement strategies (diaphyseal pins via separate incisions and metaphyseal pins via the main incision) [21], one case involved diaphyseal placement via separate incisions [14], and in two studies [24,26] the pin placement strategy was not specified. The lower frequency of separate femoral incisions, together with anatomical factors, may partly explain the lower incidence of these complications at the femur. Conversely, tibial pin placement required separate incisions in 87.5% of cases; in 12.5% the pin placement was not specified [26].

In the included studies, specific pathogens were not reported. All cases were successfully managed with oral antibiotic therapy; only two cases described by Le Brun et al. [26] required hospitalization for intravenous antibiotic administration.

Analysis of published case reports identified fracture as the predominant complication following pin placement. Of the 14 fractures reported, displaced injuries constituted 71.4%, most commonly exhibiting transverse or short-oblique configurations. All fractures were documented postoperatively, with a mean interval from surgery to fracture of 8.5 weeks. Management was dictated by fracture morphology and stability: eight patients underwent antegrade intramedullary nailing, three underwent open reduction and internal fixation with plate and screws, two were managed nonoperatively with protected weight-bearing, and one received combined intramedullary nailing with supplemental plate fixation.

Among the case series and cohort studies included, fractures had an incidence of 0.16% (24/15,004). Twenty-nine percent of fractures occurred intraoperatively; of these, six tibial fractures (cortical flecks or lucencies at pin sites) did not require modification of the standard postoperative protocol [20] (in one case, treatment was not reported), and one occult tibial fracture [21] was associated with delayed rehabilitation due to persistent pin-site pain.

Of the postoperative fractures (ranging from 4 to 12 weeks), seven were low energy [21,30], two were stress fractures [24,48], and the mechanism was not reported in eight cases. Management consisted of intramedullary nailing in six cases, ORIF in four, conservative treatment with brace and protected weight-bearing in two, not reported in four, and one revision of the tibial component with a long stem [27].

The 4–12-week onset may reflect a subacute mechanical failure process: initial cortical damage from pin insertion leads to microcrack accumulation and propagation under cyclical loading. This process is likely amplified by stress concentrations at eccentric or metaphyseal pin sites, reduced cortical cross-section, or large pin diameters. These are speculative mechanistic considerations that warrant targeted biomechanical and prospective clinical investigation.

Regarding associated comorbidities, increased body mass index (BMI) and osteoporosis emerged as potential predisposing factors.

In the present analysis, BMI data were available for eight patients who sustained postoperative pin-related fractures; the mean BMI was 32.9 kg/m^2^ (class I obesity), suggesting a potential association between increased mechanical loading and fracture risk. Osteoporosis status was not reported in five cases; eight patients were documented as not having osteoporosis. Only one patient had confirmed osteoporosis—treated with denosumab—with a lumbar spine DEXA T-score of −1.6 and multiple fragility fractures of the thoracic and lumbar spine [41]. These findings indicate that osteoporosis alone may not be a primary determinant of pin-related fracture risk.

Other comorbidities were uncommon: hypertension was recorded in two patients (one of whom also had chronic kidney disease and hyperlipidemia), and comorbidity data were unavailable for one patient.

**Figure 2:**
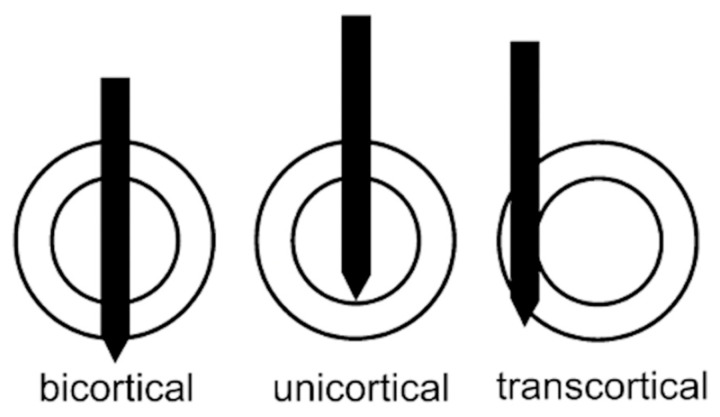
Three possible pin penetrations in diaphyseal bone. (Reproduced from Jung HJ, Jung YB, Song KS, Park SJ, Lee JS. Fractures associated with computer-navigated total knee arthroplasty. A report of two cases. J Bone Joint Surg Am. 2007 Oct; 89[10]: 2280-4.) [44].

Bicortical fixation was employed in eight patients and unicortical fixation in one patient; pin configuration was unreported in three cases. In two additional cases, planned bicortical fixation resulted in inadvertent transcortical placement intraoperatively, highlighting technical challenges in achieving accurate pin positioning (Figure 2). These observations suggest that bicortical fixation may increase fracture risk, although the literature is inconsistent. Owens et al. [29] recommend avoiding bicortical fixation, despite only two fractures being reported across their series and other retrospective studies. Conversely, Koutserimpas et al. [21] reported two fractures associated with monocortical fixation and found no statistically significant differences between fixation methods. In the Beldame et al. [30] cohort, the presence of at least one transcortical pin appeared to correlate with increased fracture risk, and Kamara et al. [14] explicitly advised against both juxta-articular and transcortical pin placement.

Analysis of fracture location showed that, in retrospective and cohort studies, most fractures occurred at the metaphyseal level. Case-report evaluation revealed a predominance of femoral involvement, comprising 12 of 14 fractures (85.7%), evenly split between diaphyseal (six cases) and distal femoral (six cases) fractures; only two fractures (14.3%) involved the tibial diaphysis. These data suggest increased susceptibility of the femur, particularly its distal segment, to pin-related fractures. Pin diameter has been proposed as a potential risk factor: Bonutti et al. [17] identified larger pin diameter as a possible contributor, and Desai et al. [20] reported five of six fractures in groups using larger-diameter pins; however, this association did not reach statistical significance and was characterized as a trend rather than a definitive risk factor. In the present case-report series, pin-diameter data were available for most patients (3.2 mm, n = 6; 3.0 mm, n = 1; 5.0 mm, n = 2; 2.5 mm, n = 1; 2.8 mm, n = 2; 3.3 mm, n = 1; unreported, n = 1). Overall, no statistically significant association between pin diameter and fracture occurrence was observed.

A variety of other, far less frequent pin-related complications were reported among the included studies: 4 cases of delayed wound healing [20,21], 1 exostosis [21], 5 instances of pin-site redness or bleeding [29], 2 cases of osteomyelitis [21,36], one suture abscess [14], 4 cases of pin-site irritation [23], and 2 vascular complications [25,37], as well as 1 case of myositis ossificans [34].

Most of these pin-related complications resolved with a short course of oral antibiotics or with no treatment, yielding favorable outcomes. Berning et al. described the first case of proximal tibial osteomyelitis related to pin placement [36]: after an initial course of antibiotics, a recurrence occurred at 6 months, requiring surgical debridement and prolonged antibiotic therapy, which resulted in a good outcome. In the second case included in the study [21], surgical debridement and 45 days of antibiotic therapy were required. Thermal injury, percutaneous (non-incisional) pin insertion, and transcortical placement—which increases thermal stress—appeared to be associated with an increased risk of this complication. Two vascular complications were reported: injury to a branch of the superficial femoral artery, requiring surgical evacuation of the hematoma and fixation with two screws [36], and injury to a branch of the anterior tibial artery, requiring endovascular treatment [25]. In Gulhane’s case report [37] the femoral pin was placed anteriorly and as proximally as possible. Alba [51] measured distances between estimated femoral pin locations and neurovascular structures, demonstrating that the profunda femoris and femoral artery are at increased risk with more proximal pin placement. Abdul-Jabar et al. reported a case of myositis ossificans at a femoral pin site that required surgical excision and resulted in a good outcome. Although more commonly observed after total hip arthroplasty, this complication has also been described following knee arthroplasty [34].

Mechanical pin-related complications were also reported: 5 pin-site dislodgements [29] and 7 pin breakages [19,32]. Pin dislodgement resulted in the interruption of the robotic procedure. Of the seven pin breakages, the procedure was completed in two cases, while the outcome/management was not reported in five. Although these events may reflect technical error, it is noteworthy that in St-Mart’s prospective case series [32] the operator had no prior robotic experience, whereas in Todesca’s prospective randomized study [19] procedures were performed by an experienced robotic surgeon.

This systematic review has inherent limitations: despite mitigation efforts, including independent literature searches by two investigators and cross-referencing of identified manuscripts, relevant articles may have been missed—particularly because only PubMed/MEDLINE was searched, so studies indexed exclusively in other databases or the grey literature could be absent—and the predominance of retrospective, low-level evidence among included studies further constrains the certainty of incidence estimates. Furthermore, the lack of registration in a public protocol registry may increase the risk of bias, and the results should be interpreted in light of this limitation. Future updates should therefore extend the search to Embase, Scopus and trial registries to ensure greater comprehensiveness.

## 5. Conclusions

The overall incidence of pin-related complications observed in this study from the non-case-report series was 0.95%, lower than the 1.4% reported in the literature [15]. Although modest, these figures are clinically relevant considering the continual increase in robotic knee surgery. Pin placement remains largely dictated by surgeon preference, and no conclusive data exist regarding the safest pin locations or fixation methods. Despite the low incidence, these complications should be disclosed and discussed with the patient during informed consent.

Further research is needed to elucidate patient-specific risk factors for pin-related complications and the safest pin placement configuration.

## Figures and Tables

**Table 1:** Characteristics of the included studies.

Author	Study Design	Level of Evidence	N°. of Institution	Study Period
Desai (2023) [20]	Retrospective cohort study	III	single	2017–2021
Koutserimpas (2025) [21]	Retrospective cohort study	III	single	2021–2024
Kamara (2017) [14]	Retrospective case series	IV	single	2013–2015
Weaver (2024) [22]	Prospective cohort	IV	single	2021–2023
Vermue (2022) [23]	Retrospective case series	IV	single	2018–1029
Held (2021) [24]	Retrospective cohort study	III	single	2017–2019
Lonner (2019) [25]	Retrospective case series	IV	single	2008–2017
LeBrun (2025) [26]	Retrospective cohort study	III	single	2016–2023
Khakha (2015) [27]	Retrospective case series	IV	single	2003–2012
Stetzer (2023) [28]	Retrospective cohort study	III	multi	2018–2022
Owens (2010) [29]	Retrospective case series	IV	single	2003–2007
Beldame (2009) [30]	Retrospective case series	IV	single	2003–2007
Todesca 2016 [19]	Prospective randomized study	II	single	2008–2009
Yun (2021) [31]	Retrospective cohort study	III	single	2017–2019
St Mart (2021) [32]	Prospective case series	IV	single	2015–2016
Del Saz (2025) [33]	Case report	V	single	2020
Abdul-Jabar (2013) [34]	Case report	V	single	2008
Wysockic (2008) [35]	Case report = 2	V	single	NR
Berning (2011) [36]	Case report	V	single	NR
Gulhane (2013) [37]	Case report	V	single	NR
Li (2008) [38]	Case report	V	single	2006
Aliya G. Feroe (2022) [39]	Case report	V	single	NR
Ossendorf (2006) [40]	Case report	V	single	2006
H. E. Skibicki (2021) [41]	Case report	V	single	NR
Lee (2025) [42]	Case report = 2	V	single	NR
Matt Blue (2018) [43]	Case report	V	single	NR
Peter Bonutti (2008) [17]	Case report = 2	V	single	NR
Ho-Joong Jung (2007) [44]	Case report = 2	V	single	NR

**Table 2:** Patient demographics and operative details from case reports.

Study	Mean Age (YR)	Sex F/M	BMI kg/m^2^	FU Months	RA vs. CAN	Model Brand	Pin Fixation	Tibial Pin Location, N° and Trajectory	Femoral Pin Location, N° and Trajectory	Pin Diameter (mm)	Procedure Type
Aliya G. Feroe 2022 [39]	67	F	33.3	NR	RA	NR	Bicortical	NR	diaphysis, 2 pins	3.3	TKA
Ossendorf 2006 [40]	65	F	37.2	12	CAN	NR	Bicortical	proximal tibia, 2 pins	distal femur, 2 pins	2.5	TKA
H. E. Skibicki 2021 [41]	77	F	NR	3	RA	Stryker Mako system (Stryker, Kalamazoo, MI, USA)	Bicortical	2 pins from anteromedial to posterolateral	diaphysis, 2 pins, from anterolateral to posteromedial	3.2	TKA
Lee 2025 [42]	67 69	F F	20.8 29.9	3 12	RA RA	CUVIS-Joint CUVIS-Joint (Curexo Inc., Irvine, CA, USA)	NR NR	Diaphysis, 2 pins Diaphysis, 2 pins	Diaphysis, 2 pins Diaphysis, 2 pins	3.2 3.2	TKA TKA
Matt Blue 2018 [43]	60	F	NR	6	CAN	NR	Unicortical	Diaphysis	Diaphysis	NR	TKA
Peter Bonutti 2008 [17]	71 77	F F	42 27	NR NR	CAN CAN	Stryker 3.2 system Stryker 3.2 system (Stryker, Kalamazoo, MI, USA)	Bicortical Bicortical	NR NR	distal femoral shaft, 1 pin, anterior to posterior; distal femoral shaft, 1 pin, anterior to posterior	5 5	TKA TKA
Ho-Joong Jung 2007 [44]	70 64	F F	NR NR	6 NR	CAN CAN	Kolibri computed tomography-free knee navigation system (BrainLAB, Munich, Germany) and Ci software (DePuy) were used; Kolibri computed tomography-free knee navigation system (BrainLAB, Munich, Germany) and Ci software (DePuy) were used.	Bicortical (incidental transcortical) Bicortical (incidental transcortical)	proximal tibia, 2 pins proximal tibia, 2 pins	distal femur, 2 pins distal femur, 2 pins	2.8 2.8	TKA TKA
Del Saz 2025 [33]	76	F	36	12	RA	NR	NR	proximal tibia, 2 pins	metaphysio-diaphyseal zone, 2 pins	3.2	TKA
Abdul-Jabar 2013 [34]	72	F	35	36	CAN	B-Braun OrthoPilot^®^ (B. Braun Aesculap, Tuttlingen, Germany)	NR	NR	NR	NR	TKA
Wysocki 2008 [35]	46 77	F F	NR	NR	CAN CAN	NR	Bicortical Bicortical	in the middle one third of the anteromedial diaphysis, 2 pins. In the middle one third of the anteromedial diaphysis, 2 pins.	at the junction of the middle and distal third of the diaphysis, 2 pins, anterior to posterior; at the junction of the middle and distal third of the diaphysis, 2 pins, anterior to posterior	3.2 3.2	TKA TKA
Berning 2011 [36]	62	M	NR	1 + 2 weeks first time and 5 after second surgery	CAN	NR	Bicortical	NR	NR	3	TKA
Gulhane 2013 [37]	58	M	NR	6	CAN	Brainlab (Brainlab AG, Feldkirchen, Germany)	Bicortical	Mid diaphysis into the subcutaneous surface of the tibia	anteriorly, “as proximally as the tourniquet will allow”	3	TKA
Li 2008 [38]	53	F	37	NR	CAN	Navigation 2.0, Stryker (Stryker, Mahwah, NJ)	Bicortical	tibial shaft, 2	10 cm above the tibiofemoral joint line, 3 pins	3	TKA

**Table 3:** Pin-related complications: management strategies and clinical outcomes from case reports.

Study	Complication	Intraop/Postop	Time to Complication (wk)	Location	Treatment	Outcome
Aliya G. Feroe 2022 [39]	Fracture, displaced, transverse	Postop	12	Femur (shaft)	Intramedullary Nail	After the procedure, the patient was able to return to her normal activities.
Ossendorf 2006 [40]	stress fracture	Postop	12	Distal femur	Unloading	At 1 yr, FU healed
H. E. Skibicki 2021 [41]	Fracture, displaced, oblique	Postop	8	Femur (shaft)	Statically locked antegrade cephalomedullary nail	3 months after the nail, the patient was able to ambulate
Lee 2025 [42]	Fracture, minimally displaced, transverse Fracture, minimally displaced, transverse	Postop Postop	8 7	Tibia (shaft) Femur (shaft)	ORIF using a narrow, limited-contact dynamic compression plate; ORIF using a broad, limited-contact dynamic compression plate	After 3 months, the patient resumed her activities and had full knee ROM; After 6 months, the patient resumed her activities and had full knee ROM
Matt Blue 2018 [43]	Fracture, displaced, oblique	Postop	6	Femur (shaft)	Intramedullary nailing	At 6 months, the patient was pain free, ambulating without issue
Peter Bonutti 2008 [17]	Fracture, displaced Fracture, displaced	Postop Postop	9 12	Distal femur Distal femur	Retrograde femoral nail Retrograde femoral nail	The patient was diagnosed with occult infection of the TKA 7 months postoperatively. The patient underwent successful two-stage debridement and reimplantation revision TKA; uneventful recovery
Ho-Joong Jung 2007 [44]	Fracture, displaced, transverse Fracture, nondisplaced, stress	Postop Postop	6 8	Femur (junction of the distal shaft and metaphysis) Tibia (left proximal pin hole)	ORIF with a locking compression plate and cancellous autograft; avoid weight-bearing activity for 4 weeks	Uneventful recovery Uneventful recovery
Del Saz 2025 [33]	Fracture, displaced, oblique	Postop	8	Displaced periprosthetic femoral metaphysio-diaphyseal fracture	Combined long cephalomedullary femoral Anterograde nail and lateral femoral plating	Satisfactory functional outcomes but still requiring assistance of a cane for walking outside home
Abdul-Jabar 2013 [34]	Myositis ossificans	Postop	2	Where the femoral marker pin during surgery had been placed	Open mini-arthrotomy (excision)	The patient remained asymptomatic with a knee range of movement of 0–111°
Wysocki 2008 [35]	Fracture, displaced, transverse (AO A3). Fracture, displaced, transverse (AO A3).	Postop Postop	10 9	Femoral diaphysis Femoral diaphysis	Statically locked antegrade intramedullary nail Statically locked antegrade intramedullary nail	The fracture healed uneventfully The fracture healed uneventfully
Berning 2011 [36]	Osteomyelitis (staphylococcus aureus)	Po stop	2 + 5 days	Distal tibial pin track	First time antibiotics (19 days postop), second time (6 months from surgery) open washout and surgical debridement and longterm antibiotics	After his oral antibiotics were discontinued, the patient continued to feel well systemically. Physical examination showed no pretibial swelling and a range of motion from 5° of fixed flexion to 110° of flexion
Gulhane 2013 [37]	Quadriceps hematoma and active bleeding into femoral canal	Intraop/postop	3 days	Femoral pin insertion	On day 10 hematoma was evacuated and two 14 mm unicortical screws were inserted into the femur at the site of the bleed to tamponade the bleed. At 2 weeks post presentation, manipulation under anesthesia for ongoing stiffness. Flexion of 0–90° was obtained but due to persistent swelling a short course of diazepam was prescribed	Improvement since the second operation and had no further complications at the final follow up 6 months later
Li 2008 [38]	Periprosthetic fracture with total displacement	Postop	4	Femoral supracondylar area	Intramedullary nail	NR

**Table 4:** Patient demographics and operative details from randomized trial, case series and cohort studies.

Study	N° of Patients	Mean Age (YR)	Sex F/M	BMI kg/m^2^	FU Months	RA vs. CAN	Model Brand	Pin Fixation	Tibial Pin Location and N° and Trajectory	Femoral Pin Location and N° and Trajectory	Pin Diameter (mm)	Procedure Type
Desai 2023 [20]	177 LPD 190 SPD 367 TOT	71.4 LPD 68.1 SPD	LPD M 52 (29.4%) F 125 (70.6%) SPD M 72 (37.9%) F 118 (62.1%)	LPD 30.2 SPD 30.4	3	RA	LPD Navio (Smith and Nephew, London, UK) SPD ROSA (Zimmer Biomet, Warsaw, IN, USA)	bicortical	four fingerbreath distal to tibial tuberosity, 2 pins	metadiaphyseal region, 2 pins, anterior to posterior	4.5 3.2	TKA
Koutserimpas 2025 [21]	651 unicortical 319 bicortical 970 TOT	median age 70	F 528 54.4%	median 27.6	minimum 2	RA	MAKO (Stryker, Kalamazoo, MI, USA)	bicortical vs. unicortical	15 cm below joint line perpendicular to medial surface of the tibia	bicortical: 10 cm above upper patellar pole 30–35° medial to the midline unicortical metaphyseal 6 cm above joint line medial to lateral	Femur 4 Tibia 3.2	TKA UKA
Kamara 2017 [14]	621 UKA 2PFA	NR	NR	NR	minimum 12	RA	MAKO RIO (Stryker, Kalamazoo, MI, USA)	bicortical	diaphyseal, 2 pins	2 diaphyseal	4	UKA PFA
Weaver 2024 [22]	367	67 + −18	M 112 (30.52%) F 255 (69.48%)	NR	minimum 12	RA	CORI Surgical System (Smith & Nephew plc, Watford, UK)	bicortical	proximal tibia through two separate incisions, 2 pins	distal femur through arthrotomy, 2 pins	4	TKA
Vermue 2022 [23]	386	70.4 +−9	M 34.7% F 65.3%	30 + −10	NR	RA	MAKO (Stryker, Kalamazoo, MI, USA)	NR	diaphyseal, 2 pins	2 pins	Femur 4 Tibia 3.2	TKA
Held 2021 [24]	111	70	M 45 (33%) F 76 (67%)	30.1	24	RA	NAVIO™ imageless surgical system (Smith & Nephew, Memphis, TN, USA)	NR	diaphyseal, 2 pins	NR	NR	TKA
Lonner 2019 [25]	1064	NR	NR	NR	91% 3 m FU	RA	MAKO (Stryker, Kalamazoo, MI, USA) and NAVIO (Smith & Nephew, Memphis, TN, USA)	NR	NR	NR	NR	UKA
LeBrun 2025 [26]	1004 all outside (35%) 1056 femoral intraincisional tibial extraincisional (37%) 820 all inside (29%) TOT 2880	63.6	TOT M 1431F1449	31.4	minimum 12	RA	MAKO (Stryker, Kalamazoo, MI, USA) and CORI Smith & Nephew, Andover, MA, USA)	NR	NR	NR	NR	TKA
Khakha 2015 [27]	1596	NR	NR	NR	NR	CAN	Styker navigation system (Stryker, Kalamazoo, MI, USA)	bicortical	metadiaphyseal, 1 pin	metadiaphyseal, 1 pin	5	TKA
Stetzer 2023 [28]	2343	66 (21–90)	M45% F55%	NR	3 m 100%; 1655 70.6% 12 mesi	RA	2211 94.4% MAKO (Stryker, Kalamazoo, MI, USA), 132 5.6% ROSA (Zimmer Biomet, Warsaw, IN, USA)	bicortical	metaphyseal, 2 pins	metaphyseal, 2 pins	4	TKA
Owens 2010 [29]	984	NR	NR	NR	12	CAN	NR	unicortical	diaphyseal, 2 pins	metaphyseal, 2 pins, lateral to medial	NR	TKA
Beldame 2009 [30]	385	73.2 (65–79) reported only for complication cases	¼ reported only for complication cases	32.6 reported only for complication cases	3 reported only for complication cases	CAN	NAVITRACK (Orthosoft, Montreal, QC, Canada)	bicortical and at least 1 transcortical in all fractured patients	diaphyseal, 1 screw for the first 9-month study period diaphyseal, 2 pins for the remainder of the study period	initially and until 2006 on the femoral metaphysis; thereafter pins were placed on the diaphysis; 1 screw for the first 9-month study period, then 2 pins;	NR	TKA
Todesca 2016 [19]	225	75.3 (65–90)	M65 F165	NR	77	CAN	Amplivision (Amplitude, Valence, France)	NR	NR	NR	NR	TKA
Yun 2021 [31]	1702TKA 901UKA TOT 2603	67 (57–81)	F	30.6 (20–45)	minimum 12	RA	Mako (Stryker, Kalamazoo, MI, USA)	bicortical 1571 (60%) vs. unicortical 1032 (40%)	bicortical: diaphyseal, 2 pins, anteromedial to posterolateral unicortical: metaphyseal, 1 pin, anteromedial to posterolateral	bicortical: diaphyseal, 2 pins, anterior to posterior unicortical: epiphyseal, 2 pins through main incision, medial to lateral	4	TKA and UKA
St Mart 2021 [32]	100	64.4 + −7.9	M 44 (51.8%) F 41(48.2)	30.6 + −5.3	21 + −4	RA	Mako (Stryker, Kalamazoo, MI, USA)	bicortical	metaphysis	metaphysis	NR	UKA

LPD = large pin diameter; SPD = small pin diameter; PFA = patellofemoral arthroplasty.

**Table 5:** Pin-related complications: management strategies and clinical outcome from randomized trial, case series and cohort studies.

Study	Complication	Intraop/Postop	Time to Complication (Week)	Location	Treatment	Outcome
Desai 2023 [20]	5 intra- and 1 post-fracture, 7 pin-site infections/drainage 2 pin-site delayed wound healing cases	fracture 5 intraoperative (4LDP 2.8% e 1 SPD 0.5%)	5 intraoperative 6 < 4 1 > 4 < 8 2 > 8	13 tibiae 1 femur (fracture) 1 unspecified (delayed wound healing)	infection = antibiotic treatment fracture = 1 postop ORIF//5 intraop (cortical flecks or lucencies between pin sites) no modification of postop protocol	infection resolved
Koutserimpas 2025 [21]	fracture delayed wound healing superficial wound infection tibial osteomyelitis exostosis	postop	fracture 1 after 12 weeks; 1 after 16 weeks	fracture: all metaphyseal femur	fractures: 1 splinted 1 ORIF	good exostosis; no treatment; good outcome
Kamara 2017 [14]	1 suture abscess 3 pin-site infections	postop	NR	all tibial	oral antibiotics	good
Weaver 2024 [22]	1pin-site fracture 1 pin-site superficial infection	postop	fracture 4 weeks	distal femur	ORIF	good
Vermue 2022 [23]	1 stress fracture	postop	NR	tibia	NR	good
Held 2021 [24]	3 superficial infections 1 stress fracture	postop	NR	infection not specified stress fracture tibia	NR	NR
Lonner 2019 [25]	1 fracture (0.09%) 4 pin-site irritation cases (0.3%) 1 pseudoaneurysm (0.09%)—branch of tibialis anterior artery	postop	1 fracture 4 pin-site irritation/infection cases 1 pseudoaneurysm 8 weeks	NR	fracture = bracing and protective weight bearing pin-site irritation/infection oral antibiotics pseudoaneurysm endovascular treatment	good
LeBrun 2025 [26]	41 tibial pin-site wound complications 3 femoral pin wound site complications	postop	NR	41 tibial 3 femoral	oral antibiotics in only 2 cases, readmission and intravenous antibiotics	NR
Khakha 2015 [27]	1 fracture 17 pin-site superficial infections	postop	fracture 12 weeks pin-site infection 6 weeks	all tibia	fracture = revision TKA with long stem infection= antibiotics	good
Stetzer 2023 [28]	2 pin-site fractures	intraop and postop	tibial intraop femoral postop fracture 5 days after	1 intraop fracture tibia 1 postop fracture femur	NR	NR
Owens 2010 [29]	12 pin-site infections 5 pain redness or bleeding cases 5 pin-site dislodgements	postop and intraop and pin dislodgement	1	tibial	infection = oral antibiotics pain or redness = no treatment or local analgesics pin dislodgement = computer assisted procedure discontinuation	good
Beldame 2009 [30]	5 femoral pin-site fractures	postop	14.2 (7–21)	all femoral 4 diaphyseal 1 metaphyseal	3 intramedullary nailing, 2 ORIF	union was observed within 3 mo for all five cases; range of motion was equivalent to or greater than before the fracture, except in one case where a 5-degree loss of flexion was observed
Todesca 2016 [19]	5 pin breakages	intraop	NR	NR	NR	NR
Yun 2021 [31]	3 femoral pin-site fractures only in BD group	postop	12 weeks	femoral	intramedullary femoral rodding	NR
St Mart 2021 [32]	2 pin breakages	intraop	NR	tibial	NA	RA procedure was continued

## Data Availability

No new data were created or analyzed in this study.

## Supplementary Materials

The following supporting information can be downloaded at: https://www.mdpi.com/article/10.3390/jcm15103793/s1, Table S1: PRISMA checklist. Reference [52] are cited in the Supplementary Materials.

## Abbreviations

The following abbreviations are used in this manuscript:

TKA	total knee arthroplasty
CAN	computer-assisted navigation
RA	robotic assistance
UKA	unicompartmental knee arthroplasty
